# Modelling sexually deceptive orchid species distributions under future climates: the importance of plant–pollinator interactions

**DOI:** 10.1038/s41598-020-67491-8

**Published:** 2020-06-30

**Authors:** Spyros Tsiftsis, Vladan Djordjević

**Affiliations:** 1 Department of Forest and Natural Environment Sciences, International Hellenic University, 1st km Dramas-Microchoriou, 66100 Drama, Greece; 2 Global Change Research Institute, Academy of Science of the Czech Republic, Bělidla 986/4a, 603 00 Brno, Czech Republic; 3 Faculty of Biology, Institute of Botany and Botanical Garden, University of Belgrade, Takovska 43, 11 000 Belgrade, Serbia

**Keywords:** Biodiversity, Climate-change ecology, Ecological modelling

## Abstract

Biotic interactions play an important role in species distribution models, whose ignorance may cause an overestimation of species' potential distributions. Species of the family Orchidaceae are almost totally dependent on mycorrhizal symbionts and pollinators, with sexually deceptive orchids being often highly specialized, and thus the interactions with their pollinators are expected to strongly affect distribution predictions. We used Maxent algorithm to explore the extent of current and future habitat suitability for two Greek endemic sexually deceptive orchids (*Ophrys argolica* and *Ophrys delphinensis*) in relation to the potential distribution of their unique pollinator (*Anthophora plagiata*). Twelve climate change scenarios were used to predict future distributions. Results indicated that the most important factors determining potential distribution were precipitation seasonality for *O. argolica* and geological substrate for *O. delphinensis*. The current potential distribution of the two orchids was almost of the same extent but spatially different, without accounting for their interaction with *A. plagiata*. When the interaction was included in the models, their potentially suitable area decreased for both species. Under future climatic conditions, the effects of the orchid-pollinator interaction were more intense. Specifically, *O. argolica* was restricted in specific areas of southern Greece, whereas *O. delphinensis* was expected to become extinct. Our findings highlighted the significant role of plant–pollinator interactions in species distribution models. Failing to study such interactions might expose plant species to serious conservation issues.

## Introduction

Climate change is currently affecting species in various ways and the prediction of its impacts is a major scientific challenge. The effective conservation of species requires the availability of suitable and often large datasets^1–3^. However, adequate species distribution data are usually lacking, and in many cases, these are of low quality and resolution. Under these constraints, species conservation actions and habitat management schemes cannot be sufficiently applied^4,5^. Furthermore, the collection of such data (i.e., species records) usually requires large amounts of resources (e.g., time, funds and personnel), that are often not available^6^. Specific algorithms have been developed, during the last two decades, to overcome such problems. These are best known as Species Distribution Models (SDMs) and are being used to solve various ecological or biodiversity problems (e.g., in reserve selection or in the identification of suitable habitats) in a wide range of organisms^4,7–9^.

In studies that use SDMs to identify potentially suitable areas in space and time, species distribution data are linked to environmental variables^7^. Climatic are among the environmental variables more frequently used, due to the great importance of climate in determining species distribution at different scales, even over large geographical areas^10^. Moreover, the availability of climatic data allow for examining numerous future climatic scenarios and consequently for studying the effects of these predicted future conditions on species' geographic range. However, it is well known that species distribution and abundance is not only determined by abiotic factors but also by other ones^11–13^.

The potentially suitable area for species whose existence is exclusively dependent on other organisms (e.g., plant–pollinator interaction) is expected to be overestimated when SDMs are used without taking into consideration these interactions. As a result, biotic interactions are increasingly used directly in the SDMs or in combination with their outcomes^14–17^.

The family Orchidaceae is one of the richest and most fascinating among the families of the plant kingdom, consisting of 736 genera^18^ and approximately 28,500 species^19^. Their distribution is not only driven by climate or other site characteristics (e.g., geology) but also by their interactions with specific biotic factors (e.g., mycorrhizal fungi, pollinators)^20–22^. Many species of this family are disappearing worldwide^23^, mostly due to habitat loss and factors such as climate change and the resulting shifts in species distributions^24,25^. Furthermore, orchids could also be negatively affected by biotic factors such as the concurrent reduction of their pollinators. These negative effects are expected to be exacerbated by climate change during the twenty-first century.

A considerable number of orchids are pollinated by specific pollinators^26^. According to Tremblay^26^, 62% of the studied orchids had a single pollinator and this percentage varied in the different subfamilies of the Orchidaceae. The most characteristic orchid pollination system where species have a high plant pollinator specificity is sexual deception^27,28^. This pollination system characterizes the European genus *Ophrys* and the species *Orchis galilaea* (Bornm. and M. Schulze) Schltr., two African *Disa* species (Jersáková et al.^29^, and references therein), more than 150 orchid species—classified in at least nine genera—in Australia^28^, as well as six genera of Central and South America^29,30^. Sexual deception by orchids imposes a high level of specialization, as insect pheromones are usually species-specific. Moreover, a large proportion of *Ophrys* species pollinators are solitary bees belonging to the oligolectic group that specializes in a particular pollen species^31^. Specialization varies from species attracted by several pollinator taxa to species pollinated by only one pollinator species. Orchids' specialized pollinators often require specific conditions (e.g., specific nesting sites), further denoting the fragile plant–insect interactions^31,32^. Due to the high pollinator specificity, it is expected that these orchids cannot form reproductive populations in areas where their pollinators are not present. As a result, although they can spread through seed dispersal and colonize areas far away from their stabilized populations (some terrestrial orchids are known to have colonized areas c. 250 km away from their nearest known populations, whereas seed dispersal over distances of 5–10 km seems to be very common^33^), they will only be able to survive for a number of years and finally they will disappear.

SDMs have been widely used to predict the potential distribution of orchid species at the global, European or regional scale (e.g.^5,16,34–40^). Although most of these studies used orchids per se and were focused on species ecology and/or conservation, only one of them explored plant–pollinator co-occurrence and how the predictions of SDMs were affected by these interactions^16^. Their study focused on the current distribution of several orchids, but the results of SDMs were not projected to future climates. Orchids and their pollinators might react in different ways to global warming. This could have serious conservation consequences for the dependent species (orchids) due to the possibility of future divergence between the potential distributions of the plants and their pollinators. We did not find any study on the effects of plant–pollinator interactions on future orchid distribution under climate change scenarios in the literature.

Based on this background, the aim of the present study was to explore how orchids' potential distribution was affected by plant–pollinator interactions under current and future climatic conditions. Thus, the major objectives were (a) to compare orchids’ projections based on a set of environmental predictors and (b) to compare orchids’ projections with the projections of an insect pollinator. Specifically, we hypothesized that the potential distribution (under current and future climatic conditions) of two *Ophrys* taxa (*O. argolica* and *O. delphinensis*), would be affected by their common and unique pollinator—*Anthophora plagiata*^41^—and consequently the predicted distribution of the pollinator would restrict the potential distribution of these two *Ophrys* taxa.

## Results

The results of the Jackknife test about variable importance are presented in Table 1. Precipitation seasonality (Bio15) was the most important variable for *O. argolica*, and geological substrate for *O. delphinensis*. The distribution of their pollinator, *A. plagiata*, was mostly explained by annual precipitation (Bio12). Contrary to the most important variables, geological substrate, precipitation seasonality (Bio15) and isothermality (Bio3) respectively, contributed the least to the distribution of the three species.

**Table 1:** Results of the Jackknife test of environmental importance in the development of Maxent models.

	*Anthophora plagiata*		*Ophrys argolica*		*Ophrys delphinensis*	
	Without variable	With only variable	Without variable	With only variable	Without variable	With only variable
Bio1	92.38	21.26	97.75	19.58	95.30	28.07
Bio2	90.89	13.47	97.83	5.07	98.18	3.28
Bio3	94.20	17.50	98.01	22.89	95.58	8.52
Bio12	72.56	52.46	90.45	31.45	94.01	19.57
Bio15	93.59	28.77	72.81	74.16	98.41	13.88
Geol	–	–	98.90	11.20	48.61	57.34

Values represent percentages in relation to the total training gain of the respective models.

*Bio1* annual mean temperature, *Bio2* mean diurnal range, *Bio3* isothermality, *Bio12* annual precipitation, *Bio15* precipitation seasonality, *Geol* geological substrate.

**Figure 1:**
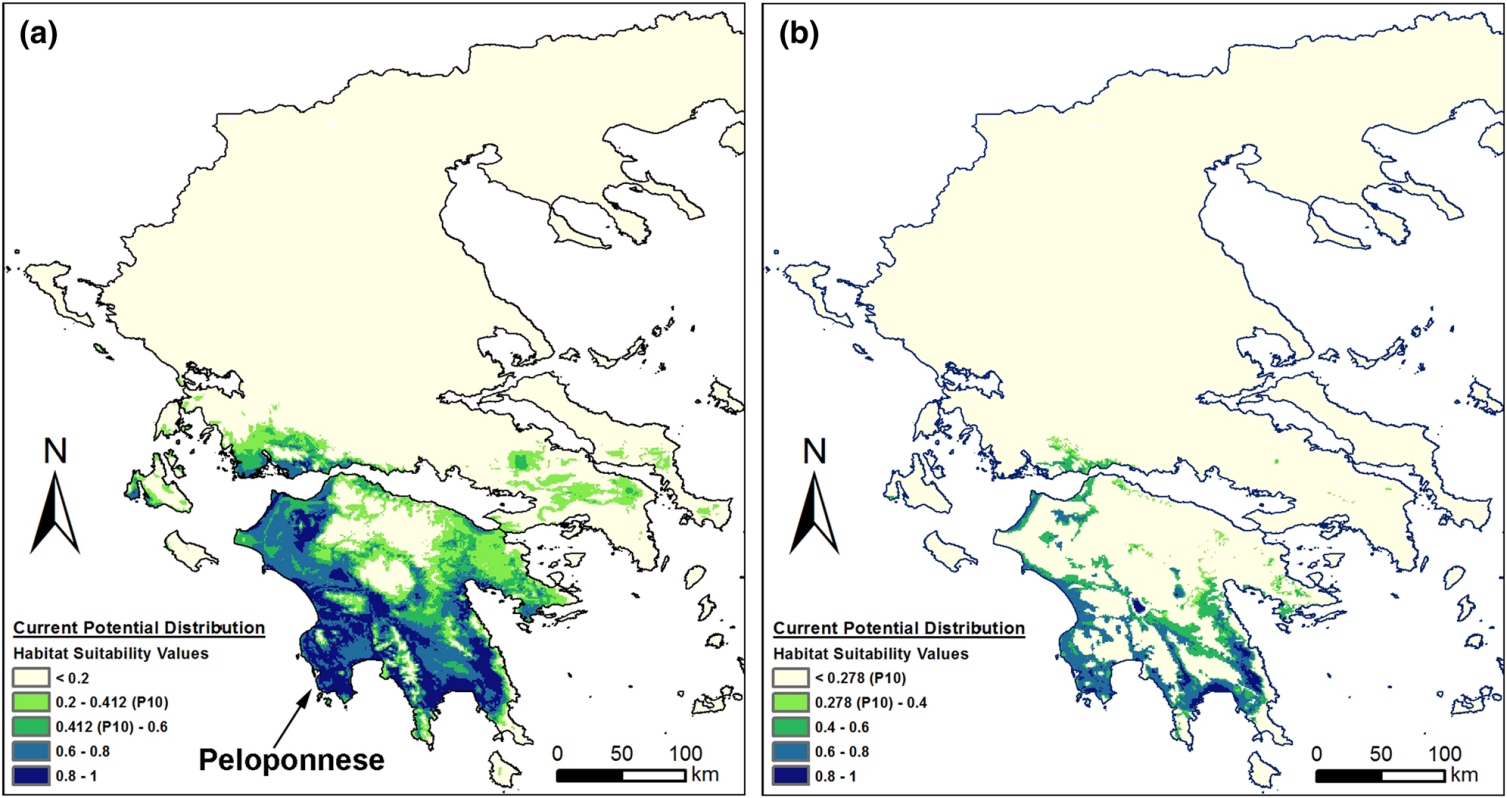
Current potential distribution (**a**) of *Ophrys argolica*, and (**b**) of *Ophrys argolica* × *Anthophora plagiata* co-occurrence model. The maps were generated in ArgGis (version 10.1, www.esri.com).

**Figure 2:**
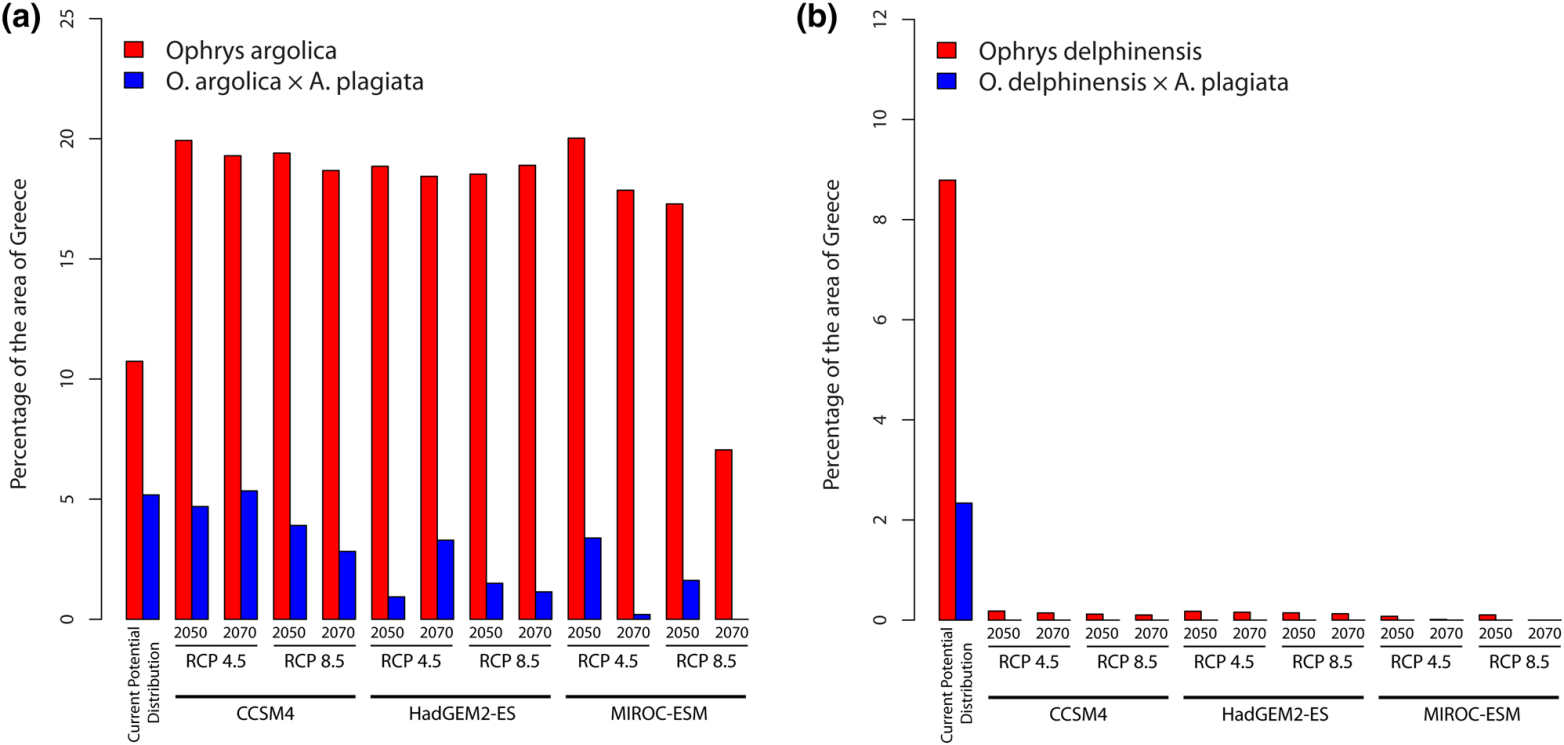
Percentage of the Greek area where each species was distributed under current and future climatic conditions; (**a**) *Ophrys argolica* and *O. argolica* × *A. plagiata*, and (**b**) *Ophrys delphinensis* and *O. delphinensis* × *A. plagiata*.

*Ophrys argolica*, per se, had its optimum in almost the whole area of the Peloponnese except for the north-central parts (Fig. 1a). However, when its current potential distribution was combined with the modelled distribution of *A. plagiata*, its potentially suitable area was reduced, mostly restricted in areas of lower altitude and close to the sea (Fig. 1b), whereas the percentage of the area of Greece that was potentially suitable for this species fell from 11 to c. 7% (Fig. 2a).

Under most of the future climatic scenarios, the potential distribution of *O. argolica* was greatly increased compared to the current potential distribution, reaching c. 20% of the total area of Greece (Fig. 2a, Supplementary Information 3), and only areas towards the highest mountain tops of the Peloponnese seemed unsuitable. Contrary to this spatial pattern, the potential distribution under the MIROC-ESM climate change model tended to be expanded to the western part of Greece (Supplementary Information 3). In general, under almost all the climatic scenarios, the Representative Concentration Pathways (RCPs) and the time periods, the potentially suitable area for *O. argolica* was retained rather stable as the percentage of the Greek area found to be suitable for *O. argolica* ranged from 17.55 to 20.32% (Fig. 2a, Supplementary Information 3). On the other hand, in only one case (scenario: MIROC-ESM; RCP: 8.5; year: 2070) the potentially suitable area for the studied species dropped down to 7.4% of the Greek area (Fig. 2a).

**Figure 3:**
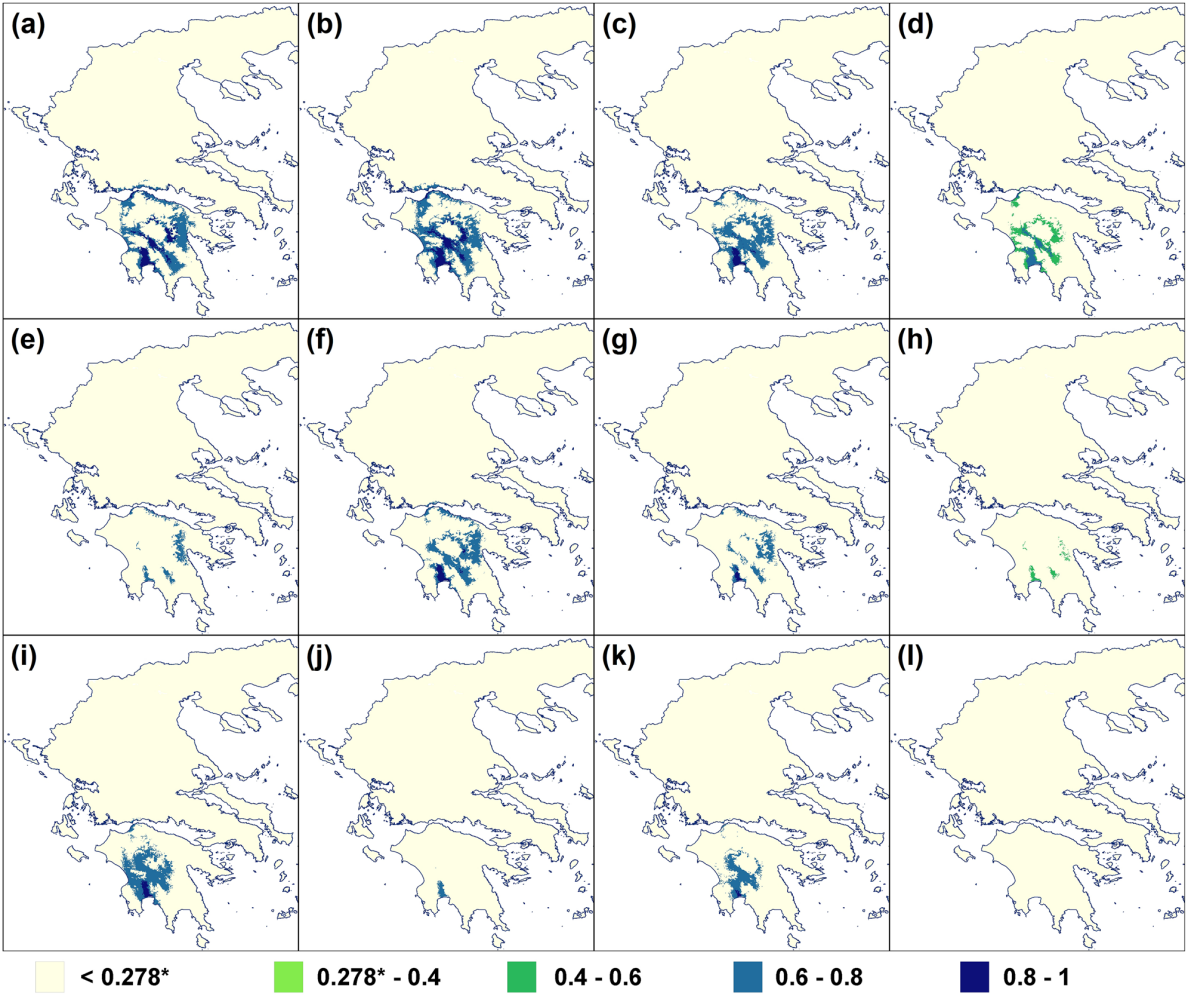
Future potential distribution of *Ophrys argolica* × *Anthophora plagiata* co-occurrence model. (**a**)–(**d**) CCSM4 [(**a**) RCP 4.5, year 2050; (**b**) RCP 4.5, year 2070; (**c**) RCP 8.5, year 2050; (**d**) RCP 8.5, year 2070]. (**e**)–(**h**) HadGEM2-ES [(**e**) RCP 4.5, year 2050; (**f**) RCP 4.5, year 2070; (**g**) RCP 8.5, year 2050; (**h**) RCP 8.5, year 2070]. (**i**)–(**l**) MIROC_ESM [(**i**) RCP 4.5, year 2050; (**j**) RCP 4.5, year 2070; (**k**) RCP 8.5, year 2050; (**l**) RCP 8.5, year 2070]. The 10th percentile training presence threshold (P10) is presented with an asterisk (*). The maps were generated in ArgGis (version 10.1, www.esri.com).

The results of the co-occurrence model (*O. argolica* × *A. plagiata*) were more complex compared to those of *O. argolica* when studied alone. Under two climate change models (CCSM4 and HadGEM2-ES) and a medium greenhouse gas emission scenario (RCP: 4.5), the potential distribution of *O. argolica* was increasing from 2050 to 2070 (Figs. 2a, 3). In all the other cases, its potential distribution was decreasing from 2050 to 2070, whereas the species is expected to become extinct in 2070 according to the MIROC-ESM climate change model (RCP: 8.5) due to the low habitat suitability values for *A. plagiata* (Supplementary Information 2).

**Figure 4:**
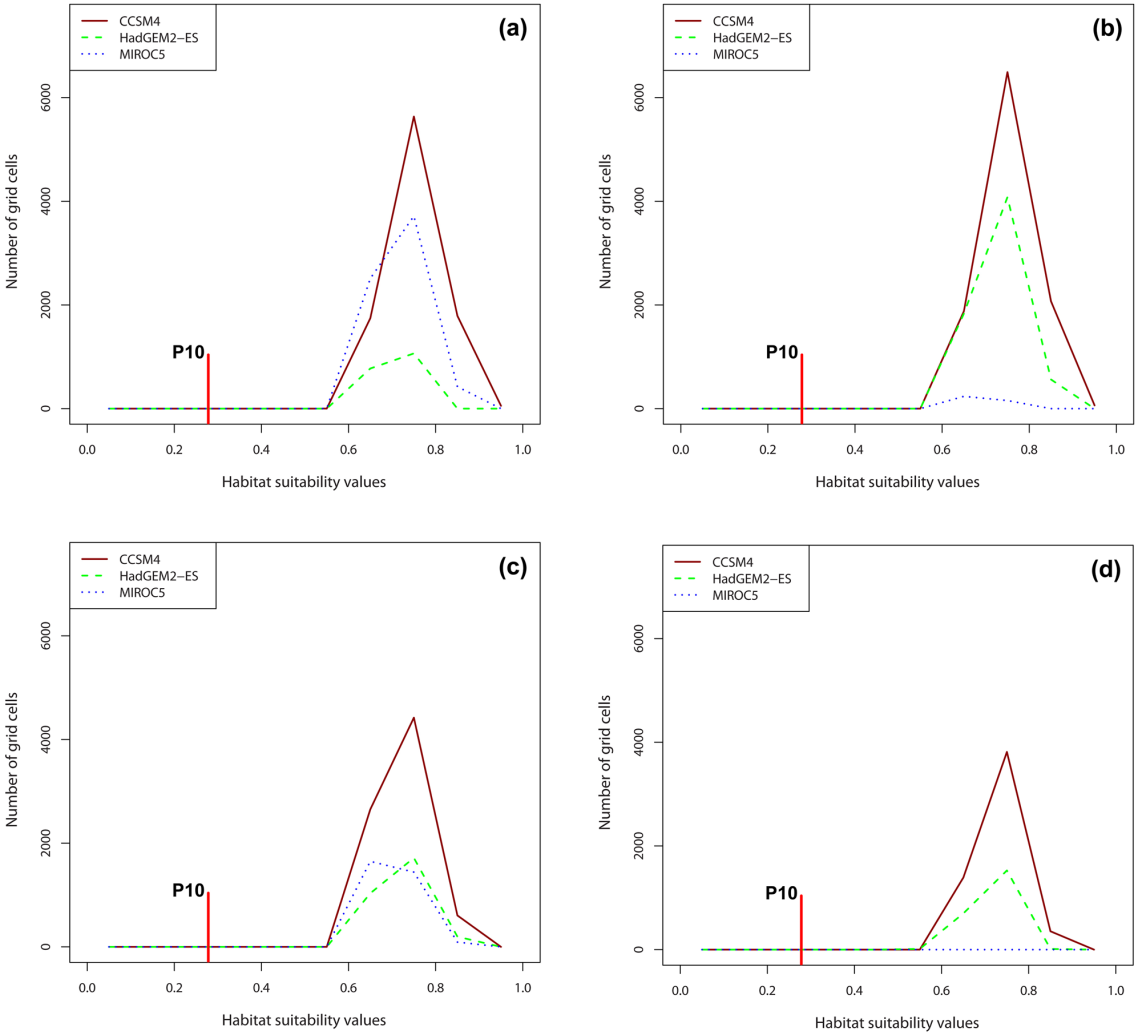
Habitat suitability values of the species co-occurrence model *Ophrys argolica* × *Anthophora plagiata* under the three climatic scenarios, in relation to the number of grid cells; (**a**) RCP 4.5, year 2050, (**b**) RCP 4.5, year 2070, (**c**) RCP 8.5, year 2050, (**d**) RCP 8.5, year 2070.

The number of grid cells along the classes of habitat suitability values for the co-occurrence model of *O. argolica* × *A. plagiata* is shown in Fig. 4. The majority of grid cells had high suitability values, above 0.6 for almost all the climatic scenarios. Moreover, the number of grid cells above the 10th percentile training presence threshold (P10) of species co-occurrence (P10 = 0.278) did not present extensive fluctuations in the cases of the CCSM4 and HadGEM2-ESM climate change models. On the other hand, the number of grid cells under the MIROC-ESM climate change model was strongly differentiated among RCPs and time periods. Specifically, c. 4,000 grid cells had values above 0.6 in 2050 (RCP: 4.5), whereas on the contrary, in 2070 under the maximum emission scenario (RCP: 8.5) all grid cells values corresponded to non-suitable areas.

**Figure 5:**
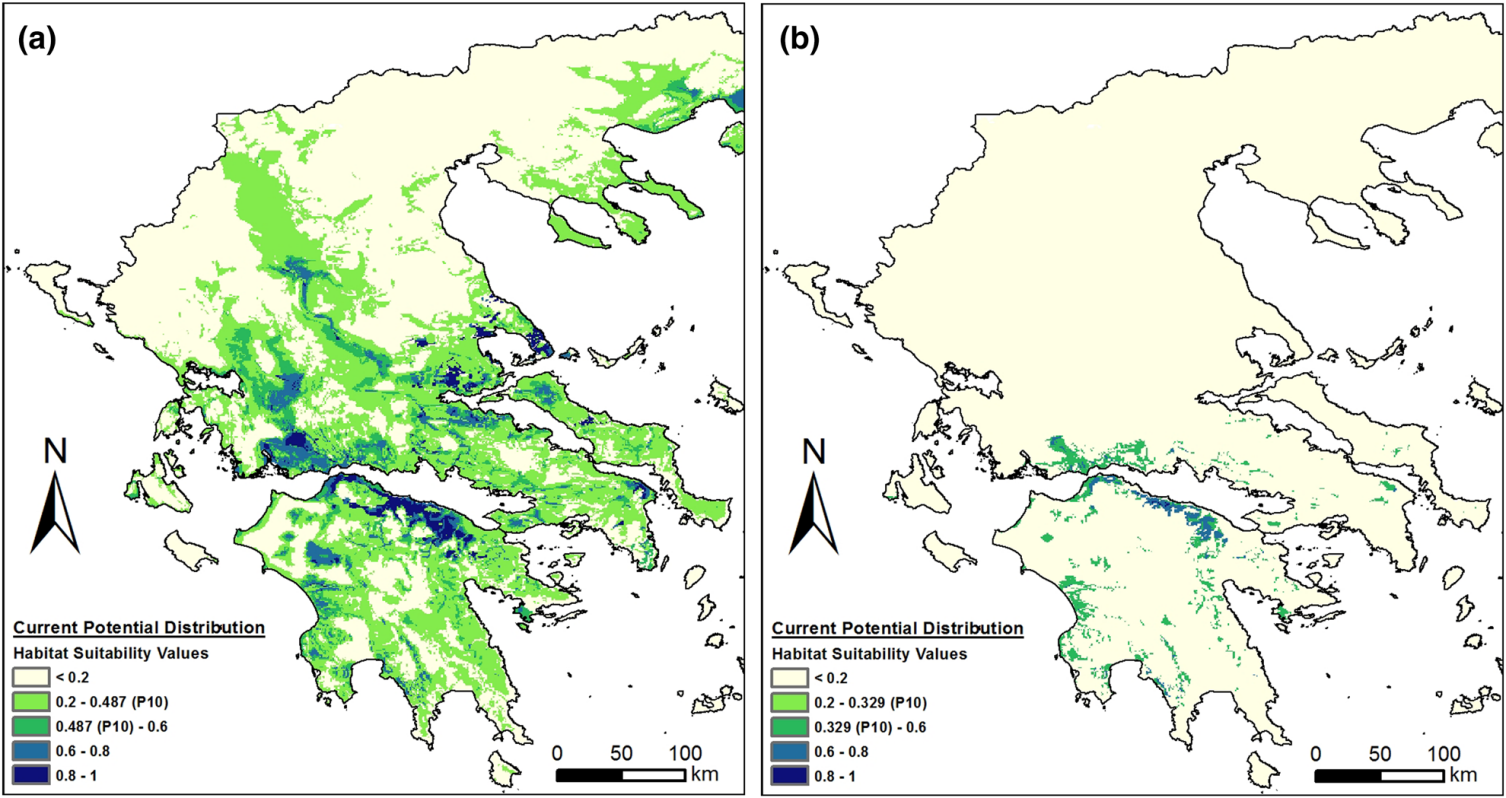
Current potential distribution of (**a**) *Ophrys delphinensis*, and (**b**) *Ophrys delphinensis* × *Anthophora plagiata* co-occurrence model. The maps were generated in ArgGis (version 10.1, www.esri.com).

According to the results of the Maxent model under the current climatic conditions, the most suitable areas for *O. delphinensis* were located in the northern part of Peloponnese, in the south-west part of Sterea Hellas and the central-east part of the mainland Greece (Fig. 5a). Moreover, although the model identified potentially suitable areas in the northern part of Greece, these areas could not be colonized by the specific orchid for biogeographical reasons. However, when taking into consideration the potential distribution of its pollinator (*A. plagiata*) the areas of high suitability values were greatly reduced, mostly restricted to the northern part of the Peloponnese and the southern part of Sterea Hellas (Fig. 5b).

**Figure 6:**
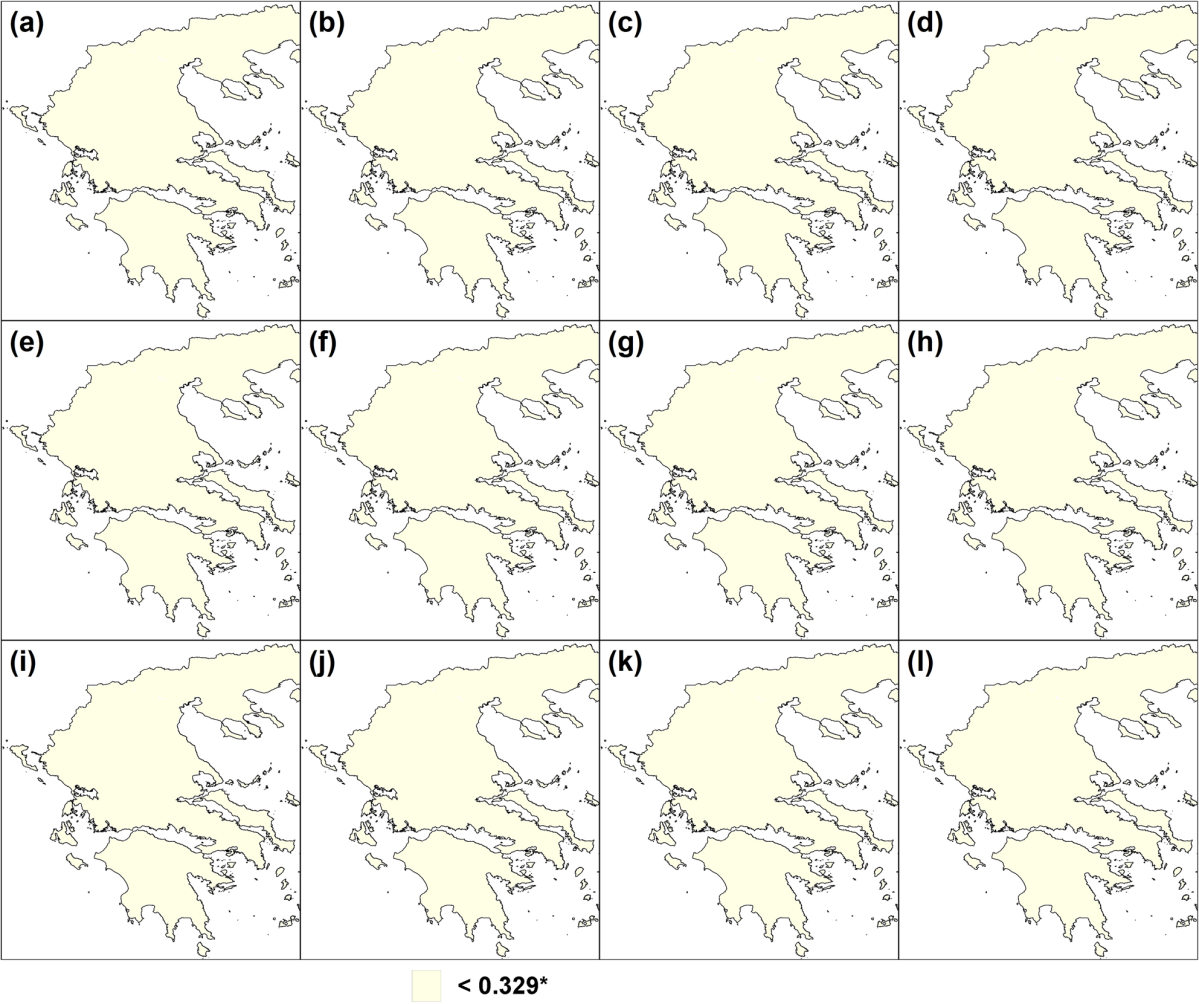
Future potential distribution of *Ophrys delphinensis* × *Anthophora plagiata* co-occurrence model. The areas where the grid cells with habitat suitability value above the 10th percentile training presence threshold are indicated with the red ellipses. (**a**)–(**d**) CCSM4 [(**a**) RCP 4.5, year 2050; (**b**) RCP 4.5, year 2070; (**c**) RCP 8.5, year 2050; (**d**) RCP 8.5, year 2070]. (**e**)–(**h**) HadGEM2-ES [(**e**) RCP 4.5, year 2050; (**f**) RCP 4.5, year 2070; (**g**) RCP 8.5, year 2050; (**h**) RCP 8.5, year 2070]. (**i**)–(**l**) MIROC_ESM [(**i**) RCP 4.5, year 2050; (**j**) RCP 4.5, year 2070; (**k**) RCP 8.5, year 2050; (**l**) RCP 8.5, year 2070]. The 10th percentile training presence threshold (P10) is presented with an asterisk (*). The maps were generated in ArgGis (version 10.1, www.esri.com).

Surprisingly, under almost all the future climatic scenarios, the most suitable sites for the distribution of *O. delphinensis* were restricted in the central-east part of mainland Greece, as well as in the north-east part of Peloponnese, whereas in these areas the habitat suitability values fell below the P10 threshold (Supplementary Information 4). However, even this restricted distribution was not congruent at all with the future potential distribution of *A. plagiata* (Supplementary Information 2), demonstrating that the species will not be able to survive (Figs. 2b, 6; Supplementary Information 5).

*Anthophora plagiata* was overlapping to a greater degree with *O. argolica* (*I *statistic = 0.453) compared to *O. delphinensis* (*I *statistic = 0.186), under current climate conditions (Table 2). It is worth mentioned that in two out of three climate change models (CCSM4 and HadGEM2-ES), niche overlap between these two species (*O. argolica* and *A. plagiata*) was increasing from 2050 to 2070 under the medium greenhouse gas emission scenario (RCP: 4.5). On the other hand, according to the third model (MIROC-ESM) niche overlap between *O. argolica* and its pollinator was decreasing from 2050 to 2070. Contrary to *O. argolica*, niche overlap values between *O. delphinensis* and *A. plagiata* under future climatic conditions demonstrated that their potential distributions were not overlapping (in all comparisons).

**Table 2:** I statistic between the two studied orchid species and their pollinator under current and future climatic conditions.

			*Ophrys argolica*		*Ophrys delphinensis*	
***Anthophora plagiata***	Current climatic conditions		0.453		0.186	
			2050	2070	2050	2070
	CCSM4	RCP 4.5	0.511	0.551	0	0
		RCP 8.5	0.470	0.410	0	0
	HadGEM2-ES	RCP 4.5	0.241	0.449	0	0
		RCP 8.5	0.305	0.266	0	0
	MIROC-ESM	RCP 4.5	0.438	0.113	0	0
		RCP 8.5	0.327	NA	0	NA

## Discussion

Conservation actions often require knowledge of species' realized niche, which is defined as a subset of species' fundamental niche under specific restrictions (e.g., biotic interactions)^4,42^. Contrary to the realized niche, fundamental niche is independent of any biotic interaction and is referred to the potential distribution of a species, which is determined by the SDMs on the basis of the environmental conditions of the presence locations.

In our study, the distribution of the two orchids seemed to be determined by different factors. In the case of *O. argolica*, its distribution was mostly determined by the effect of precipitation, whereas geological substrate was not important. The opposite trend had been found in the case of *O. delphinensis*. This could be due to the fact that the studied orchids have different preferences regarding the geological substrate of the sites where they have been recorded so far. Although *O. delphinensis* is mostly found in limestones, *O. argolica* is more generalist and can be found in a great variety of substrates^43^. Moreover, contrary to other orchids whose existence strongly depends on vegetation (e.g.^5^), both orchids can be found in several types of habitats (e.g., grasslands, scrubs, forests), even in anthropogenic habitats, such as olive groves which are common in the southern parts of Greece^43^.

Although proper selection of the environmental variables used in the SDMs is of crucial importance, species interactions should not be ignored. Our results showed that biotic interactions should be considered as a key component in modelling species' distributions, because potentially suitable areas could be overestimated when they are not included in the models. Orchids depend on pollinators and mycorrhizal symbionts for their survival, and some of them present a high degree of specialization^20,22^. However, orchid mycorrhizal fungi are often present at sites where orchids are lacking, suggesting that fungi are not particularly limiting orchid distribution^22,44^. On the contrary, most terrestrial orchids can survive pollinator deficiency for only 3–10 years^32^. However, it is expected that climate change will severely affect pollinators in the future. Unfavourable future climatic conditions can either cause a great reduction in insects' populations or can change the time of their emergence, or finally, can cause a range shift towards areas of favourable climate^45,46^. In turn, these changes will cause a temporal or/and spatial mismatch between plants and their pollinators and will affect plant populations and their viability^47^. Therefore, plant–pollinator interactions could heavily affect the outcome of SDMs, especially for orchid species with high pollinator specificity.

The analysis of the current potential distribution of the two orchid species, both with and without plant–pollinator interactions, demonstrated the significance of such interactions in the outcomes of SDMs. Specifically, although the potential distribution of both orchids was almost of the same extent (10.74% for *O. argolica* and 8.79% of the area of Greece for *O. delphinensis*), after calculating habitat suitability values on the basis of their interaction with *A. plagiata*, the potentially suitable area decreased by c. 52% and c. 73%, respectively. These results are consistent with the findings of Duffy and Johnson^16^ who found that pollinators constrain the distribution of the orchids which are characterized by a highly specialized pollination system.

Our study also explored the consequences of global warming to the future potential distribution of the two studied orchids. There are several ways in which organisms may respond to climate change. Species either can adapt to the new ecological conditions, by shifting their niche, or they can move to other areas (e.g., northwards). However, the responses of species that have strong and specialized interactions with others are always very complex. Robbirt et al*.*^48^ found that *Ophrys sphegodes* and its pollinator, male bees of the species *Andrena nigroaenea*, showed phenological responses to climate warming, by reducing the frequency of pseudocopulation and, as a result, pollination success and seed production. Another eventuality is that species that interact with others can migrate and colonize different areas under the effects of climate change. In doing so, their area of co-occurrence could decrease, and in some cases the hitherto interacting species could be even spatially separated^17,49^.

In our study, the two orchids were characterized by entirely different trends under future climate change scenarios. *O. argolica* was expected to retain its distribution range under the CCSM4 climate change model when its interaction with *A. plagiata* was considered, whereas on the contrary, it was expected to narrow its range under all other scenarios. The fact that habitat suitability values of the co-occurrence model *O. argolica* × *A. plagiata* were high for a large number of grid cells under most scenarios, demonstrated that both species can co-occur successfully for a relatively extensive area, as both are characterized by high suitability values.

The worst case scenario is that species might be unable to adapt to the changing environment^50^. In our case, *O. delphinensis* was expected to become extinct in the future, as it was predicted that *O. delphinensis* and *A. plagiata* will not occur in the same areas. Although this is a pessimistic scenario, it was the simple result of an algorithm, Maxent model, which is not able to consider limitations such as species evolution and niche shift^51,52^. *Ophrys delphinensis*, as all *Ophrys* species that are highly specialized, attracts its unique pollinator (*A. plagiata*) using sexual deception. The orchid emits volatile chemical compounds similar to the pheromones of female *A. plagiata*, so attracting its pollinators who attempt copulation (pseudocopulation) with the orchid lip^31,53^. Sexual deception is an evolutionary trait of *Ophrys* taxa and Schlüter et al*.*^54^ found that small changes in the genes involved in scent production could cause the attraction of a different pollinator. This allow for the hypothesis that *O. delphinensis* could be able to attract another insect-pollinator as the result of mutations or genetic drift. The fact that *O. delphinensis* is considered as a stabilized hybrid between *O. argolica* and a taxon of the *Ophrys oestrifera* group, two taxa that are pollinated by different bees (reviewed in Ref.^43^), offers further support to this hypothesis.

Our findings suggested that the effect of the interaction of the two orchids with their unique pollinator is very important under both current and future climate conditions. Although the study dealt only with plant–pollinator interactions, future research should also focus on the potential effect of their mycorrhizal symbionts. By doing this, we will be able to explore whether orchids can shift their distribution under climate change using a more complete spectrum of environmental and biotic constraints. Given that many orchids, either in Greece or in Europe^55,56^, have declined dramatically during the twentieth century and continue to decline, better insights into the processes affecting the distribution and abundance of orchids will be of high value for conservation management and the restoration of orchid populations. This study provides information on the distribution of *Ophrys argolica* and *O. deplhinensis*, whereas can serve as a basis for other studies dealing with the potential distribution of orchid species and their pollinators. Moreover, although the orchid datasets used in the analyses are good from a quantitative and qualitative perspective, a systematic survey for the presence of *A. plagiata* in other parts of Greece could provide valuable ecological information and improve the results of the SDMs and consequently the effective conservation of the studied orchids.

## Material and methods

### Studied species

We used as case studies two Greek orchids to explore the effects of plant–pollinator interactions in species distribution modelling: *Ophrys argolica* H.Fleischm. (*O. argolica* subsp. *argolica *sensu WCSP) and *Ophrys delphinensis* O. Danesch and E. Danesch. (*O.* × *delphinensis *sensu WCSP). The two studied orchid taxa are Greek endemics and are distributed mainly to the southern mainland part of the country^43,57^. Based on their known distribution, the study area was set between 36° 05′–41° 37′ N and 19° 30′–24° 40′ E. Species of the European genus *Ophrys* usually present a high degree of pollinator specificity as many of them are being pollinated by a single pollinator^27^. Both, *O. argolica* and *O. delphinensis* are sexually deceptive orchids and are exclusively pollinated by the same pollinator, *Anthophora plagiata* (Illiger, 1806)^41,43,58,59^, an insect which is distributed in several European countries but is rather rare in the southern part of Europe^60^.

### Species distribution models

The extent of current and future habitat suitability for *O. argolica* and *O. delphinensis* in Greece in relation to the potential distribution of their unique pollinator (*A. plagiata*) was explored using Maxent software (version 3.4.1)^61,62^. Maxent is considered as an appropriate technique for modelling species distributions, even in the case of very small sample size^7,63^, and thus is the most widely used method. The model estimates species distribution based on presence-only data, using the maximum entropy principle, and computes a probability distribution based on environmental variables spread over the entire study area. However, the probability value of each grid cell corresponds to the habitat suitability value of the specific grid cell.

In total, three datasets of species occurrences were created, one for each plant and pollinator species. The distribution data for the two orchid species used in the respective models, were based on the database that was built for the purposes of the Orchid Flora of Greece project^64^. The third dataset corresponded to the occurrences of the pollinator of the two studied orchids. However, there aren't available detailed data about the distribution of *A. plagiata* in Greece (either in published sources or in GBIF—www.gbif.org). To overcome this problem, we used a combined database of orchid occurrences, consisted of the total number of records of the two studied orchids, as well as all the known records of the taxon *Ophrys olympiotissa* Paulus, also pollinated by *A. plagiata*^57^, as a surrogate. It is worth mentioned that in the vast majority of these sites, pollinated flowers of the three orchids have been detected, clearly indicating that the pollinator (*A. plagiata*) is present. So, in this way by using this approach we assumed that the three orchids and their pollinator were present in the same sites.

### Model settings and selection

Together with the species distribution data, a second input in Maxent software corresponded to the environmental variables that were used to predict the potential distribution of the studied species. Initially, 20 environmental variables were selected as predictors in species distribution modelling. Nineteen of them were bioclimatic variables and one corresponded to the geological substrate of Greece. The bioclimatic variables were obtained from the WorldClim database^65,66^ in a 30-s resolution (approximately 1 km^2^), whereas the environmental layer corresponding to the geological substrate was at a scale of 1:500,000^67^. Although the map of the geological substrate was in vector format, the layer was converted in raster format at the same resolution and extent with the layers of the bioclimatic variables. To account for multicollinearity between the 19 bioclimatic variables and avoid overfitting, Pearson correlation coefficients were calculated for all pairwise interactions, for each species. To eliminate highly correlated variables, only one (i.e. the one with the higher percent contribution and training gain) was selected among any pair of those with a correlation coefficient r >|0.75|^68,69^. Specifically, in modelling the potential distribution of the orchid species, the non highly inter-correlated bioclimatic variables and the geological substrate were used. On the contrary, in modelling the pollinator of the *Ophrys* spp. only the selected bioclimatic variables were used. Moreover, having in mind that *A. plagiata* has a rather wide distribution in Europe^60^ and we used distribution data only from the southern part of mainland Greece, a bias file was used, with which the background samples exploited by the algorithm were restricted to areas that fall within a distance (20 km) from the presence locations^70^. The idea behind that data handling was that biases in background samples will account for similar biases in presence locations^70^.

Five (5) models for each species were run with Maxent using the auto-features mode and the default settings, as suggested by Phillips and Dudík^61^. Specifically, we used 10,000 background points and we increased to 5,000 the number of maximum iterations. This was done to provide adequate time to the model for convergence. Moreover, we removed duplicate presence records to reduce the effect of spatial auto-correlation. Maxent models were run using cross-validation as a form of replication. Model performance was assessed using the Akaike information criterion (AICc) because it greatly outperforms BIC and AUC based methods, especially when sample size is small^71^.

### Climate projection models

To explore the future potential distribution of the two studied orchid species (*O. argolica* and *O. delphinensis*), as well as their pollinator (*A. plagiata*) in Greece, 12 climate change scenarios were used, corresponding to the years 2050 (average for 2041–2060) and 2070 (average for 2061–2080). Specifically, for each period (2050 and 2070), three climate change models were chosen under CMIP5. The chosen climate change models were the Hadley Earth System Model (HadGEM2-ES), the Community Change System Model (CCSM4) and the Model for Interdisciplinary Research On Climate (Earth System Model) (MIROC-ESM). For each period and each climate change model, two Representative Concentration Pathways (RCPs) were used. These three climate change models have shown good performance in simulating future climate, and as a result they have been widely used in the last decade in studies of range shifts^17,72,73^. The four available pathways differ in the greenhouse gases that are to be emitted in the future. Specifically, the RCP2.6 is a low emission scenario, RCP4.5 and RCP6.0 are medium greenhouse gas emission scenarios, whereas the RCP8.5 is a maximum emission scenario. Here we used the maximum greenhouse gas emission scenario (RCP8.5) and one out of the two medium greenhouse gas emission scenarios (RCP 4.5).

### Co-occurrence models

Except of the direct output of the Maxent models, the main aim of the present study was to identify the areas where the two studied orchids can potentially occur in relation to their pollinator. In probability theory, the probability of co-occurrence of two species (A and B) is given by the product of P(A) × P(B)^74^. However, this is accurate and correct only if species occur independently and are not interacting. In general, species distributions derived by species distribution models is assumed to be independent, and these distributions are not influenced from any ecological interaction among species^75^.

To calculate the probability of suitable environmental conditions for both species (species co-occurrence) we used only the habitat suitability values that were above the 10th percentile training presence threshold (P10) for each species separately, whereas values lower than the P10 were set to zero. Among the several thresholds that can be calculated by Maxent software, we used the 10th percentile training presence threshold, as it provides a better ecologically significant result in comparison with others^61^.

Based on the above-mentioned assumption, the probability (habitat suitability value) of orchid-pollinator co-occurrence (species co-occurrence model: plant × pollinator) can be calculated using the formula:

$$ P\left( {A_{i}^{^{\prime}} } \right) = P\left( {A_{i} } \right)\bigcap\limits_{i}^{n} {P\left( {C_{i} } \right)\,\,{\text{if}}\,{\text{and}}\,{\text{only}}\,{\text{if}}\,P(A_{i} ) \ge {\text{ P1}}0_{A} \,{\text{and}}\,P(C_{i} ) \ge {\text{ P1}}0_{C} } $$

where, *P*(*A*_*i*_*'*) is the habitat suitability value for each pair of co-occurring species (*Ophrys argolica*–*Anthophora plagiata* and *Ophrys delphinensis*–*Anthophora plagiata*, respectively), *P*(*A*_*i*_) is the habitat suitability value of species *A* for the grid cell *i* (applicable for *O. argolica* and *O. delphinensis*, respectively), *P*(*C*_*i*_) is the habitat suitability value of the pollinator (here *A. plagiata*), for the grid cell *i*, P10 corresponds to the values of the 10th percentile training presence threshold for the three studied species (*A. plagiata*: P10_*C*_ = 0.675; *O. argolica*: P10 = 0.412; *O. delphinensis*: P10 = 0.487), *n* is the number of grid cells that all the raster layers generated by MaxEnt algorithm contain.

### Niche overlap between plant–pollinator potential distribution

The Warren's I similarity statistic^76^ was used to assess the statistical similarities between the potential distribution of the two studied orchids (*O. argolica* and *O. delphinensis*) and their pollinator (*A. plagiata*) under the current climatic conditions, as well as under the future climatic scenarios. This statistic uses suitability scores and has been widely used in the past (e.g.^77,78^). It measures niche overlap between two species distribution models and its values range from 0 (no overlap between the two distributions) to 1 (identical distributions). Prior the calculation of the "*I *statistic", habitat suitability values below the P10 threshold for each species were set to zero.

All analyses were performed in R version 3.5.2 (R Foundation for Statistical Computing) using the packages 'raster', 'rgdal', 'dismo', 'ENMeval' and 'SDMTools', whereas the distribution maps were generated in ArcMap 10.1^79^.

## Supplementary information

Supplementary information

## Data Availability

Species records that were used in developing niche models are available from the corresponding author upon request.
