# Supplementary material for: Modelling sexually deceptive orchid species distributions under future climates: the importance of plant–pollinator interactions
Source: Sci Rep. 2020 Jun 30;10:10623. doi: 10.1038/s41598-020-67491-8 (PMC7327032; doi:10.1038/s41598-020-67491-8)
Supplement: Supplementary file 1 — Supplementary information [file 41598_2020_67491_MOESM1_ESM.docx]

**Modelling sexually deceptive orchid species distributions under future climates: the importance of plant-pollinator interactions**

Journal: Scientific Reports

Spyros Tsiftsis, Vladan Djordjević

**Corresponding author**: Tsiftsis Spyros; Department of Forest and Natural Environment Sciences, International Hellenic University, GR-66100 Drama, Greece, stsiftsis@for.ihu.gr


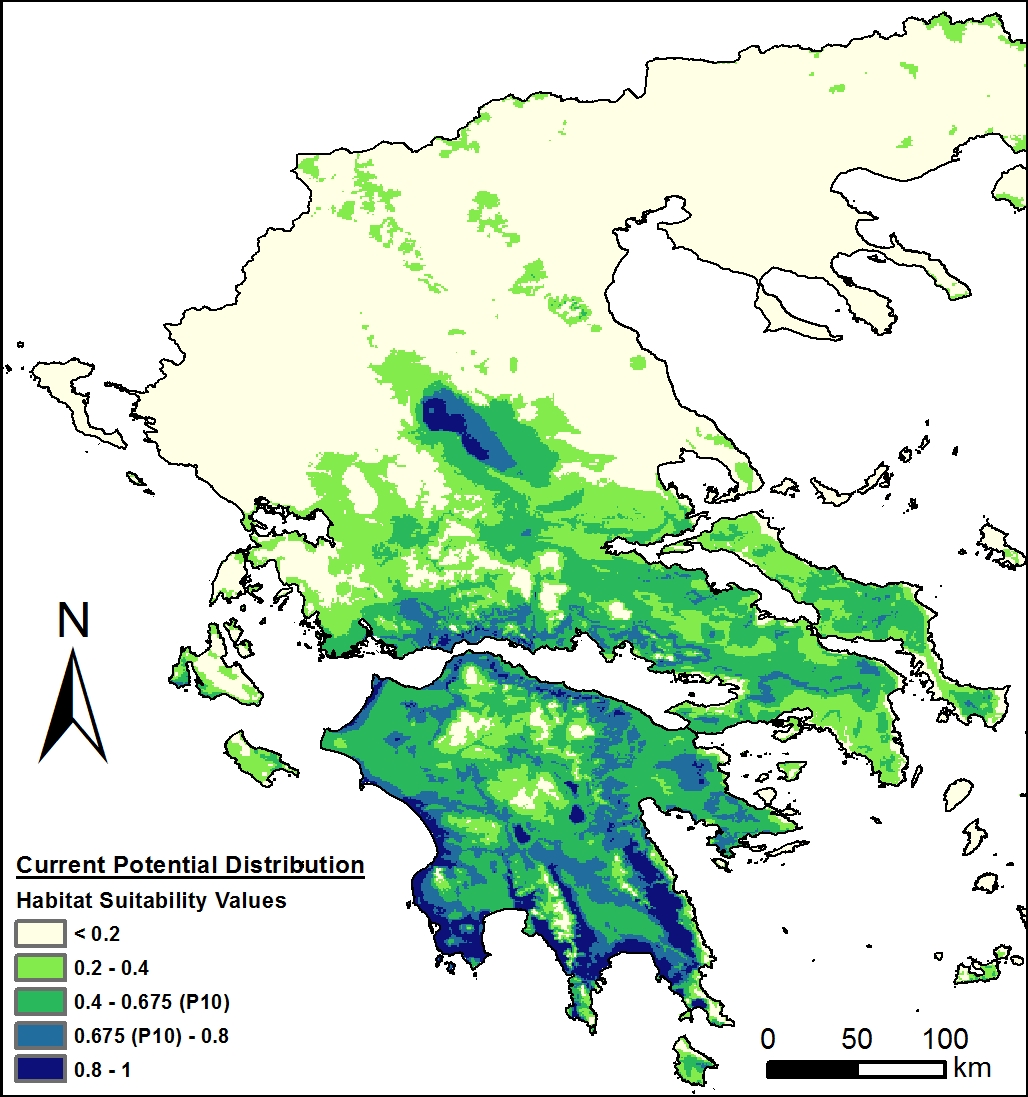


Electronic Supplementary Material 1. Current potential distribution of *Anthophora plagiata*. The maps were generated in ArgGis (version 10.1, www.esri.com).


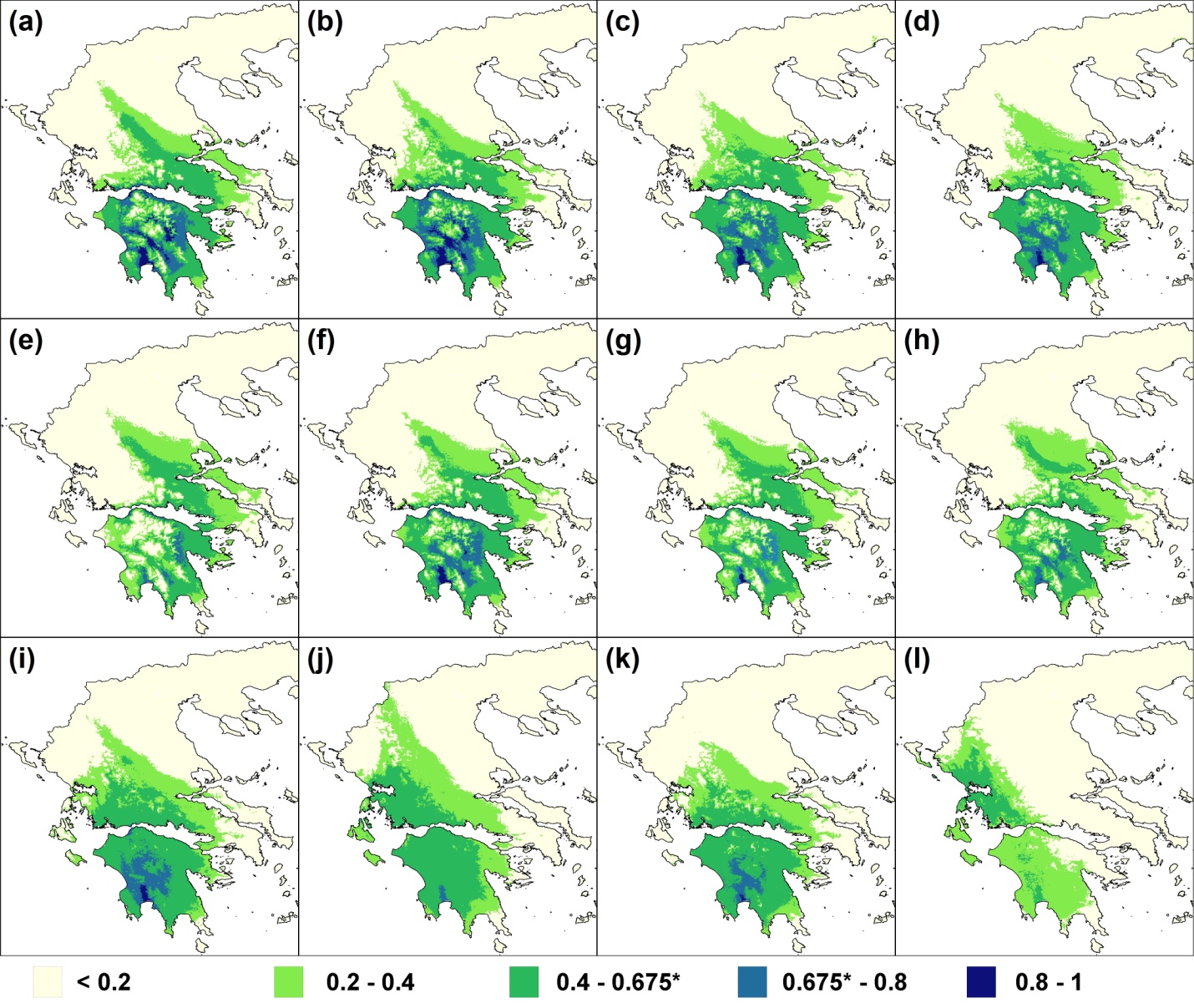


Electronic Supplementary Material 2. Future potential distribution of *Anthophora plagiata*. (a)-(d): CCSM4 [(a): RCP 4.5, year 2050; (b): RCP 4.5, year 2070; (c): RCP 8.5, year 2050; (d): RCP 8.5, year 2070]. (e)-(h): HadGEM2-ES [(e): RCP 4.5, year 2050; (f): RCP 4.5, year 2070; (g): RCP 8.5, year 2050; (h): RCP 8.5, year 2070]. (i)-(l): MIROC_ESM [(i): RCP 4.5, year 2050; (j): RCP 4.5, year 2070; (k): RCP 8.5, year 2050; (l): RCP 8.5, year 2070]. 10^th^ percentile training presence threshold (P10) is presented with an asterisk (*).The maps were generated in ArgGis (version 10.1, www.esri.com).


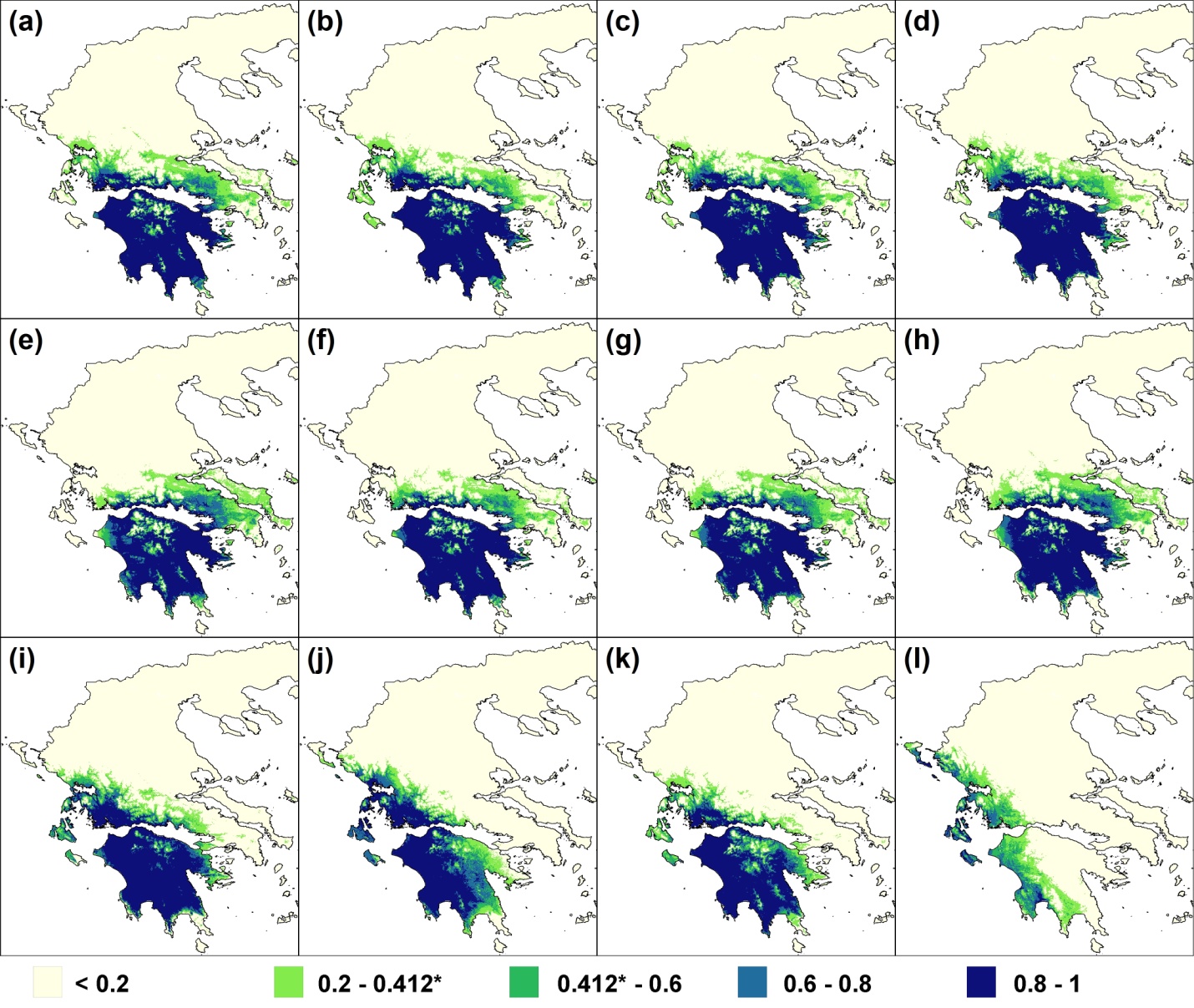


Electronic Supplementary Material 3. Future potential distribution of *Ophrys argolica*. (a)-(d): CCSM4 [(a): RCP 4.5, year 2050; (b): RCP 4.5, year 2070; (c): RCP 8.5, year 2050; (d): RCP 8.5, year 2070]. (e)-(h): HadGEM2-ES [(e): RCP 4.5, year 2050; (f): RCP 4.5, year 2070; (g): RCP 8.5, year 2050; (h): RCP 8.5, year 2070]. (i)-(l): MIROC_ESM [(i): RCP 4.5, year 2050; (j): RCP 4.5, year 2070; (k): RCP 8.5, year 2050; (l): RCP 8.5, year 2070]. The 10^th^ percentile training presence threshold (P10) is presented with an asterisk (*).The maps were generated in ArgGis (version 10.1, www.esri.com).


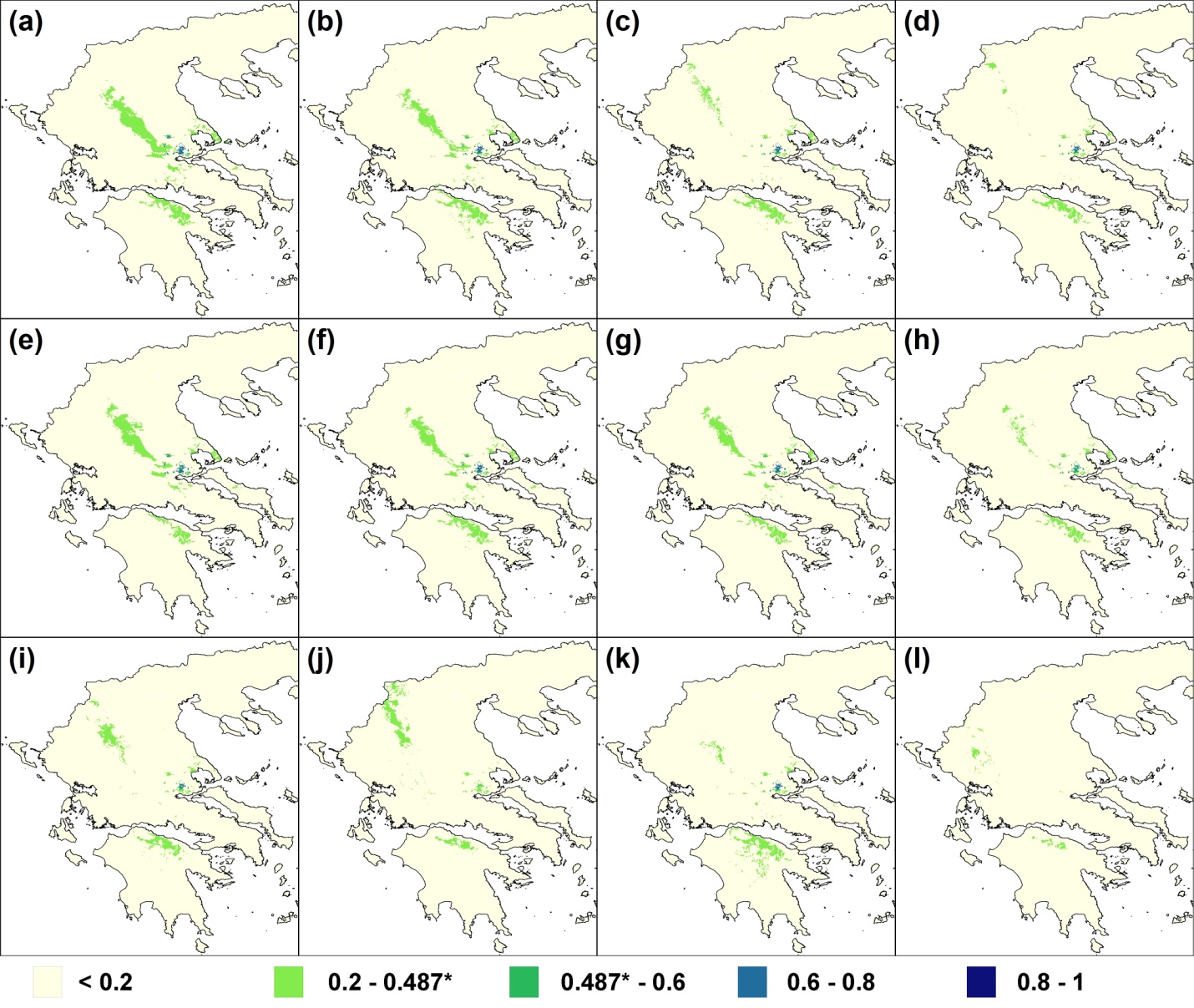


Electronic Supplementary Material 4. Future potential distribution of *Ophrys delphinensis*. (a)-(d): CCSM4 [(a): RCP 4.5, year 2050; (b): RCP 4.5, year 2070; (c): RCP 8.5, year 2050; (d): RCP 8.5, year 2070]. (e)-(h): HadGEM2-ES [(e): RCP 4.5, year 2050; (f): RCP 4.5, year 2070; (g): RCP 8.5, year 2050; (h): RCP 8.5, year 2070]. (i)-(l): MIROC_ESM [(i): RCP 4.5, year 2050; (j): RCP 4.5, year 2070; (k): RCP 8.5, year 2050; (l): RCP 8.5, year 2070]. The 10^th^ percentile training presence threshold (P10) is presented with an asterisk (*).The maps were generated in ArgGis (version 10.1, www.esri.com).

Electronic Supplementary Material 5: Number of 1×1 km grid cells with habitat suitability value above the 10^th^ percentile training presence threshold

|  |  |  | Number of grid cells above the lowest presence threshold | | | | |
| --- | --- | --- | --- | --- | --- | --- | --- |
| Climate change models | Representative Concentration Pathways | Year | *Anthophora plagiata* | *Ophrys argolica* | *Ophrys delphinensis* | *O. argolica* × *A. plagiata* | *O. delphinensis* × *A. plagiata* |
| CCSM4 | RCP 4.5 | 2050 | 9271 | 39174 | 354 | 9224 | 0 |
|  |  | 2070 | 10529 | 37925 | 283 | 10504 | 0 |
|  | RCP 8.5 | 2050 | 7697 | 38143 | 236 | 7680 | 0 |
|  |  | 2070 | 5557 | 36714 | 200 | 5557 | 0 |
| HadGEM2_ES | RCP 4.5 | 2050 | 1860 | 37060 | 346 | 1839 | 0 |
|  |  | 2070 | 6519 | 36234 | 309 | 6476 | 0 |
|  | RCP 8.5 | 2050 | 2960 | 36415 | 286 | 2947 | 0 |
|  |  | 2070 | 2252 | 37143 | 251 | 2252 | 0 |
| MIROC-ESM | RCP 4.5 | 2050 | 6650 | 39358 | 150 | 6650 | 0 |
|  |  | 2070 | 393 | 35097 | 27 | 393 | 0 |
|  | RCP 8.5 | 2050 | 3184 | 33975 | 203 | 3184 | 0 |
|  |  | 2070 | 0 | 13864 | 0 | 0 | 0 |
